# Conocimiento, actitud y percepción sobre riesgos radiológicos y uso del consentimiento informado radiológico entre los odontólogos que laboran en una universidad peruana

**DOI:** 10.21142/2523-2754-1304-2025-262.

**Published:** 2025-11-08

**Authors:** Leslie Romina Paredes-Cruz, Laura Ricardina Ramírez-Sotelo, María Eugenia Guerrero

**Affiliations:** 1 Facultad de Odontologia, Universidad Nacional Mayor de San Marcos. Lima, Peru. leslie.paredes.cruz@gmail.com Universidad Nacional Mayor de San Marcos Facultad de Odontologia Universidad Nacional Mayor de San Marcos Lima Peru leslie.paredes.cruz@gmail.com; 2 Departamento Medico-Quirurgico, Facultad de Odontologia, Universidad Nacional Mayor de San Marcos. Lima, Peru. lramirezs@unmsm.edu.pe mguerreroac@unmsm.edu.pe Universidad Nacional Mayor de San Marcos Departamento Medico-Quirurgico Facultad de Odontologia Universidad Nacional Mayor de San Marcos Lima Peru lramirezs@unmsm.edu.pe mguerreroac@unmsm.edu.pe

**Keywords:** conocimiento, actitud, percepción, radiología, dentista, knowledge, attitudes, perception, radiology, dentists

## Abstract

**Objetivo:**

Evaluar el nivel de conocimiento, actitud y percepción sobre los riesgos radiológicos y el uso del consentimiento informado en radiología bucal y maxilofacial entre los odontólogos que laboran en la Universidad Nacional Mayor de San Marcos (UNMSM), en Lima, Perú.

**Material y métodos:**

El diseño del presente estudio fue de tipo observacional, descriptivo y transversal. Se elaboró un instrumento de 25 ítems en forma de cuestionario de opción múltiple utilizando Google Forms. La primera sección incluye datos sociodemográficos y la segunda presenta la evaluación del conocimiento, actitud y percepción sobre los riesgos radiológicos y el uso del consentimiento informado (CI) en radiología bucal y maxilofacial. Para el análisis se incluyeron 89 profesionales docentes. Los datos fueron analizados por la prueba de chi-cuadrado y mediante un análisis de regresión logística, para lo cual se utilizó un nivel de significación de p ≤ 0,05.

**Resultados:**

Se encontró que el nivel de conocimiento fue alto (76,40%) y medio (23,60%), mientras que las preguntas relacionadas al órgano que requiere protección radiográfica y el conocimiento de los principios de protección radiológica fueron estadísticamente diferentes según el grado académico y años de experiencia clínica, respectivamente (p ≤ 0,05). Con respecto a la actitud y percepción, la mayoría de los participantes usan siempre o muy frecuentemente delantal de plomo (62,93%) y collar tiroideo (42,70%) en sus pacientes. La percepción presentó diferencias significativas según el grado académico y entidad clínica en la que labora (p ≤ 0,05).

**Conclusiones:**

Los resultados ofrecen una mirada representativa del conocimiento, actitud y percepciones que podrían estar presentes en otros entornos educativos y clínicos, los cuales contribuyen a generar conciencia en los odontólogos, promover el uso del CI en radiología odontológica, orientar intervenciones formativas, políticas institucionales y marcos normativos.

## Abstract (en)

**Objective:**

To evaluate the level of knowledge, attitude, and perception of radiological risks and the use of informed consent in oral and maxillofacial radiology among dentists working at the National University of San Marcos (UNMSM).

**Materials and Methods:**

The design of this study was observational, descriptive, and cross-sectional. A 25-item multiple-choice questionnaire was developed using Google Forms. The first section presented sociodemographic data, and the second section presented the assessment of knowledge, attitude, and perception of radiological risks and the use of informed consent (IC) in oral and maxillofacial radiology. Eighty-nine teaching professionals were included in the analysis. The data were analyzed using the chi-square test and logistic regression analysis, with a significance level of p≤ 0.05.

**Results:**

The level of knowledge was found to be high (76.40%) and medium (23.60%) in the sample; however, only questions related to the organ requiring radiographic protection and knowledge of the principles of radiation protection were statistically different according to academic degree and years of clinical experience, respectively (p≤0.05). With regard to attitude and perception, most participants always or very frequently use lead aprons (62.93%) and thyroid collars (42.70%) on their patients, while only perception showed significant differences according to academic degree and clinical entity in which they work (p≤0.05).

**Conclusions:**

The results offer a representative view of the knowledge, practices, and perceptions that could be present in other educational and clinical settings. These could raise awareness among dentists, promote the use of IC in dental radiology, and guide training interventions, institutional policies, and regulatory frameworks.

## INTRODUCCIÓN

La indicación de métodos de exámenes radiográficos viene siendo ampliamente utilizada en odontología. Las dosis de radiación de radiografías intraorales periapicales varían entre 3-15 μSv y entre 9-24 μSv para radiografías panorámicas ^1^. Por otro lado, una tomografía computarizada de haz cónico (TCHC) puede emitir una mayor dosis de radiación y el rango de dosis varía dependiendo del campo de visión, con lo que puede alcanzar valores desde 19 hasta 1073 μSv. Esto implica cierto grado de riesgo radiológico para los pacientes ^(1, 2)^ y produce algún efecto dañino para la salud del paciente u operador ^(3, 4)^.

Con respecto al conocimiento del paciente sobre la exposición a la radiación ionizante en los procedimientos de imágenes radiográficas médicas u odontológicas, la literatura concuerda en que los pacientes desconocen los riesgos y no tienen conciencia sobre la exposición a la radiación, y destaca la falta de comunicación con los profesionales de salud con respecto a la exposición a la radiación ^(5, 6)^. Por tanto, resulta necesario informar mejor a los pacientes sobre la exposición a la radiación a la que serán sometidos y aclarar que una información adecuada y precisa es fundamental para garantizar que el principio del consentimiento esté presente ^(5, 6)^. Una formación continua y eficaz para el personal odontológico que incluya los riesgos y beneficios sobre exámenes por imágenes para pacientes pediátricos y sus padres/tutores fueron también evaluados concluyendo que existen brechas de conocimientos y habilidades entre los residentes y médicos ^7^^-^^10^.

El uso del consentimiento informado (CI) puede variar de acuerdo con la ley estatal, la política institucional, departamental y la práctica de la comunidad local ^5^. A pesar de que los profesionales conocen la importancia del uso del CI, en la actualidad no existe un consenso claro sobre su uso ^11^. Por su parte, el uso del CI escrito es una práctica común en todo el mundo, pero en la práctica radiológica sigue siendo controversial en muchas naciones ^5^. Por este motivo, diversos autores confirman la importancia de plantear una serie unificada y definitiva de pautas para el uso del CI en los exámenes con uso de radiación ionizante ^(10, 12)^.

Al respecto, según Yurt *et al*., el conocimiento, las actitudes y los comportamientos de los dentistas sobre la protección radiológica y la obtención de imágenes dentales debe ser mejorado, y resulta de suma importancia la implantación y continuación de programas de formación de alta calidad adecuadas a las normas establecidas por la autoridad nacional de cada país ^13^. Asimismo, otros estudios refieren que, en general, el personal radiológico percibe que la exposición a bajas dosis de radiación en la vida diaria y los entornos laborales no representan ningún riesgo para la salud ^13^. Así, un mayor nivel de conocimiento de la radiación se asoció estrechamente con una menor percepción de riesgo ^(14, 15)^.

En la literatura se pueden hallar diversos estudios que evalúan el conocimiento, la actitud y la práctica aplicada a la radiología odontológica, respecto de la seguridad y la protección radiológica. Así, se encontraron estudios desde el uso de delantales de plomo ^16^; comparación entre cirujanos dentistas ^17^, estudiantes de pregrado ^18^^-^^20^, postgrado ^21^ y practicantes de odontología ^22^; dosis de radiación y los riesgos asociados ^23^; dentistas privados ^24^ y años de experiencia ^10^. Las diferencias encontradas por algunos de estos estudios fueron influenciadas principalmente por el tiempo de años de experiencia clínica, los grados académicos del profesional y las entidades en las que labora ^24^.

La literatura demuestra que las principales razones de la información inadecuada sobre los riesgos en radiología odontológica fueron el desconocimiento de las responsabilidades, la creencia de que la información no era necesaria y la preocupación por despertar temores innecesarios ^23^. Existen pocos estudios realizados en la población peruana con relación a los riesgos radiológicos y el uso de un CI en el área de radiología bucal y maxilofacial ^(25, 26)^ con aplicación de estilo KAP (*knowledge, attitude and perception*) ^27^. Se encontraron evidencias que evaluaron el nivel de conocimiento sobre protección radiológica en estudiantes e internos de odontología, y se halló que el nivel de conocimiento es intermedio ^(26, 28)^, mientras que algunos otros cuestionan el uso de un CI en el campo radiológico ^(29, 30)^. Ninguno de estos estudios incluyó como población a docentes o cirujanos dentistas con años de experiencia clínica. Sin embargo, poco se ha reportado desde el punto de vista odontológico. Por ese motivo, la presente investigación tuvo como objetivo evaluar el nivel de conocimiento, actitud y percepción sobre los riesgos radiológicos y el uso del consentimiento informado en radiología bucal y maxilofacial entre odontólogos que laboran en la Universidad Nacional Mayor de San Marcos (UNMSM) con respecto a su grado académico, años de experiencia clínica y la entidad donde desarrollan su práctica clínica.

## MATERIAL Y MÉTODOS

El trabajo de investigación de tipo observacional, descriptivo y transversal cuenta con la aprobación del Comité Institucional de Ética en Investigación del Instituto de Medicina Tropical Daniel Alcides Carrión, de la Facultad de Medicina de la UNMSM, con código de revisión CIEI-IMT-01-2021.

Para determinar el tamaño de la muestra, se realizó un muestreo no probabilístico por conveniencia que incluyó un total de 89 docentes cirujanos dentistas, de los cuales 60 eran de sexo masculino y 29, de sexo femenino, con edades entre 30 y 75 años, quienes laboran en pregrado y posgrado en la Facultad de Odontología de la UNMSM. Los docentes fueron reagrupados considerando la edad (30-40, 40-50, 50-60 y más de 60 años), el tiempo de experiencia clínica de los docentes (10-20, 20-30, 30-40 y más de 40 años), el grado académico de los participantes (magíster/especialista y doctor) y, finalmente, la entidad en la que laboran (privada o pública). 

Para la recolección de datos se utilizó un cuestionario de opción múltiple virtual mediante Google Forms, el mismo que presentaba 25 preguntas, divididas en dos secciones. La primera sección constaba de datos sociodemográficos como sexo, edad, entidad donde labora, años de experiencia clínica y grado académico. La segunda sección constaba de la evaluación del conocimiento, la actitud y la percepción sobre los riesgos radiológicos, así como el uso del CI en radiología bucal y maxilofacial. El cuestionario del nivel de conocimiento, actitud y percepción sobre los riesgos radiológicos fue creado a partir de los estudios previos en la literatura ^(19, 21)^ (tabla 1).

**Tabla 1:** Cuestionario sobre conocimiento, actitud y percepción sobre riesgos radiológicos y uso del consentimiento informado radiológico

Preguntas del cuestionario	
Conocimiento	• ¿Tiene conocimiento sobre el principio de ALARA (“Tan bajo como aceptable diagnósticamente”) o ALADA (“Tan bajo como sea razonablemente posible”)?
	• ¿Tienen las radiografías efectos secundarios en los pacientes?
	• Con respecto a la dosis de radiación ionizante: una dosis de radiación ionizante baja, pero que se aplica durante un periodo prolongado, ¿tendría riesgo para el paciente?
	• ¿Qué examen tendrá mayor dosis de radiación? ¿Dieciséis (16) radiografías dentales intraorales o una tomografía computarizada *cone beam*?
	• ¿El uso de radiografía digital requiere menor radiación que una radiografía convencional?
	• Los pacientes que han sido expuestos con anterioridad (por motivos médicos) a radiación ionizante por un largo periodo de tiempo, ¿tienen mayor riesgo de sufrir cáncer, aunque reciban dosis bajas de radiación?
	• ¿Es la sensibilidad a la radiación ionizante directamente proporcional a la edad?
	• ¿Qué órganos del cuerpo requieren protección para una toma radiográfica dental: la glándula tiroides o las gónadas?
	• ¿Cree Ud. que las radiografías constituyen una necesidad para poder dar un diagnóstico certero?
	• ¿Cómo define “consentimiento informado” en la práctica clínica?
Actitud	• ¿Usa el delantal de plomo en sus pacientes durante el examen radiológico?
	• ¿Usa collar tiroideo en sus pacientes durante el examen radiológico?
	• ¿Se ubica detrás de una barrera de plomo durante la exposición?
	• ¿Mantiene la película radiográfica en la boca de los pacientes durante la exposición?
	• ¿Hace uso del consentimiento informado en su práctica clínica diaria?
	• ¿Informa a los pacientes acerca de los riesgos de la radiación antes de realizar una toma radiológica en su práctica diaria?
Percepción	• ¿Se debería informar al paciente acerca de los riesgos de cáncer causados por radiación?
	• ¿Es necesario obtener el consentimiento informado de los pacientes ante una radiología bucal y/o maxilofacial?
	• ¿Solo se debería informar al paciente acerca de los riesgos involucrados en una toma radiográfica, mas no necesariamente obtener un documento firmado?
	• ¿Solo es necesario realizar un consentimiento informado en radiología bucal y maxilofacial en casos especiales, como niños, mujeres embarazadas y/o pacientes con quimioterapia?

Para evaluar el nivel de conocimiento se establecieron 10 preguntas, de las cuales se asignó 1 punto a cada pregunta correctamente respondida. Luego, el puntaje total fue recategorizado: de 0 a 3 puntos se consideró un nivel de conocimiento bajo; de 4 a 6 puntos, un nivel de conocimiento medio; y de 7 a 10 puntos, un nivel de conocimiento alto. Con respecto a la evaluación de la actitud, se utilizó la escala Likert de 5 opciones, donde “1” corresponde a “Siempre/ muy frecuentemente”, “2” a “Frecuentemente”, “3” a “Ocasionalmente”, “4” a “Raras veces” y “5” a “Nunca”. Finalmente, para evaluar la percepción se utilizó también la escala Likert, de 5 opciones, donde “1” corresponde a “Totalmente de acuerdo”, “2” a “De acuerdo”, “3” a “Indiferente”, “4” a “En desacuerdo” y “5” a “Totalmente en desacuerdo”. El cuestionario del uso del CI fue diseñado por dos especialistas en radiología bucal y maxilofacial, y posteriormente sometido a pruebas de validez y confiabilidad por prueba de expertos. Para determinar la confiabilidad del instrumento, se desarrolló una prueba piloto de 30 cirujanos dentistas, donde se evaluó la consistencia interna mediante el alfa de Cronbach, obteniendo un valor de α = 0,73, lo cual indica una confiabilidad aceptable. Posteriormente, el cuestionario se entregó a los docentes cirujanos dentistas que laboran en la UNMSM de manera virtual mediante correo electrónico o redes sociales WhatsApp o Messenger de Facebook.

## ANÁLISIS ESTADÍSTICO

Los datos se analizaron utilizando el programa Stata versión 17. Se realizó un análisis descriptivo en el cual se utilizaron frecuencias y porcentajes para las variables cualitativas, y medidas de tendencia central y dispersión para las variables cuantitativas. Para el análisis inferencial, las variables se sometieron a las pruebas de normalidad y, de acuerdo con eso, se sometieron a pruebas no paramétricas de chi cuadrado y un análisis de regresión logística con un nivel de significancia del 5% (p ≤ 0,05).

## RESULTADOS

El número total de participantes fue de 89, de los cuales el 67,42% fueron hombres y el 32,58%, mujeres. Con respecto a la edad de los encuestados, se encontró que la mayoría se encuentran en el rango de edad de 60 años a más (46,07%). Con respecto al grado académico, se halló que el 64,04% tienen el grado de magíster y/o especialista. Con respecto a la entidad donde laboran, se encontró que la mayoría (62,92%) laboran de manera particular o privada (tabla 2).

**Tabla 2:** Datos sociodemográficos de los participantes

Variable	Categoría	N	%
Sexo	Femenino	29	32,58
	Masculino	60	67,42
Edad	30-40 años	9	10,11
	40-50 años	15	16,85
	50-60 años	24	26,97
	60 años a más	41	46,07
Años de experiencia clínica	10-20 años	14	15,73
	20-30 años	31	34,83
	30-40 años	31	34,83
	40 años a más	13	14,61
Grado académico	Magíster/Especialista	57	64,04
	Doctor	32	35,96
Entidad donde labora	Pública	33	37,08
	Privada	56	62,92

En relación con el nivel de conocimiento de los odontólogos que laboran en la UNMSM sobre los riesgos radiológicos y el uso del CI en radiología bucal y maxilofacial, según sus años de experiencia clínica, grado académico y la entidad donde laboran, fue alto en un 76,40% y medio en 23,60%; no hubo calificativo bajo entre los encuestados. Sin embargo, no fue observada ninguna diferencia significativa entre las subcategorías de cada grupo evaluado (tabla 3).

**Tabla 3:** Nivel de conocimiento según los años de experiencia clínica, grado académico y la entidad donde laboran

	Nivel de conocimiento		Valor p
	Alto	Medio	Prueba χ²
Años de experiencia			
10-20 años	12 (13,48%)	2 (2,25%)	
20-30 años	24 (26,97%)	7 (7,87%)	0,481
30-40 años	21 (23,60%)	10 (11,24%)	
40 años a más	11 (12,36%)	2 (2,25%)	
Grado académico			
Doctor	24 (26,97%)	8 (8,99%)	0,815
Magíster /especialista	44 (49,44%)	13 (14,61%)	
Entidad donde labora			
Pública	23 (25,84%)	10 (11,24%)	0,253
Privada	45 (50,56%)	11 (12,36%)	
Total	68 (76,40%)	21 (23,60%)	

Los resultados mostraron también que, entre las preguntas relacionadas al conocimiento de los odontólogos, actitud y percepción sobre los riesgos radiológicos y el uso del CI en radiología bucal y maxilofacial, considerando los años de experiencia del profesional, la pregunta “¿Conoce el principio de ALARA o ALADA?” fue estadísticamente diferente, siendo mayor la respuesta afirmativa entre los profesionales con 20 a 30 años de experiencia, con un valor p = 0,041. En la variable grado académico, las preguntas “¿Qué órgano requiere de protección para una toma radiográfica dental?” y “¿Solo es necesario un consentimiento informado en casos especiales: como niños, mujeres embarazadas y/o pacientes con quimioterapia?” fueron estadísticamente diferentes en el grupo de Magíster/especialista, con un p = 0,038 y p = 0,001, respectivamente. Finalmente, considerando la entidad donde el profesional labora, ninguna pregunta fue respondida estadísticamente diferente entre los profesionales que laboran en entidades privadas o públicas (tabla 4).

**Tabla 4:** Distribución de las respuestas obtenidas al cuestionario de conocimiento según el grado académico de los participantes

Pregunta	Respuesta	Grado académico		Total	Valor p
		Magíster/ Especialista n.o (%)	Doctor n.o (%)		Prueba χ²
¿Conoce el principio de ALARA o ALADA?	SÍ	26 (29,21%)	18 (20,22%)	44 (49,44%)	0,336
	NO	31 (34,83%)	14 (15,73%)	45 (50,56%)	
¿Tienen las radiografías efectos secundarios en los pacientes?	SÍ	40 (44,94%)	20 (22,47%)	60 (67,42%)	0,458
	NO	17 (19,10%)	12 (13,48%)	29 (32,58%)	
Una dosis de radiación baja aplicada en período prolongado, ¿tendría riesgo para el paciente?	SÍ	49 (55,06%)	31 (34,83%)	80 (89,89%)	0,101
	NO	8 (8,99%)	1 (1,12)	9 (10,11%)	
¿Qué examen tendrá mayor dosis de radiación?	16 radiografías intraorales.	40 (44,94%)	25 (28,09%)	65 (73,03%)	0,417
	TCHC	17 (19,10%)	7 (7,87%)	24 (26,97%)	
¿La radiografía digital requiere menor radiación que una radiografía convencional?	SÍ	49 (55,06%)	25 (28,09%)	74 (83,15%)	0,343
	NO	8 (8,99%)	7 (7,87%)	15 (16,85%)	
Los pacientes que han sido expuestos con anterioridad a radiación, ¿tienen mayor riesgo de sufrir cáncer?	SÍ	46 (51,69%)	29 (32,58%)	75 (84,27%)	0,217
	NO	11 (12,36%)	3 (3,37%)	14 (15,73%)	
¿Es la sensibilidad a la radiación ionizante directamente proporcional a la edad?	SÍ	34 (38,20%)	16 (17,98%)	50 (56,18%)	0,379
	NO	23 (25,84%)	16 (17,98%)	39 (43,82%)	
¿Qué órgano requiere de protección para una toma radiográfica dental?	Glándula tiroidea	52 (58,43%)	24 (26,97%)	76 (85,39%)	0,038
	Gónadas	5 (5,62%)	8 (8,99%)	13 (14,61%)	
¿Son las radiografías necesarias para un diagnóstico certero?	SÍ	44 (49,44%)	27 (30,34%)	71 (79,78%)	0,418
	NO	13 (14,61%)	5 (5,62%)	18 (20,22%)	
¿Cómo define CI en la práctica clínica?	Conformidad del paciente...	44 (49,44%)	23 (25,84%)	67 (75,28%)	0,577
	Firma de documento de conformidad...	13 (14,61%)	9 (10,11%)	22 (24,72%)	

Se realizó un análisis de regresión logística para evaluar la asociación entre la pregunta ¿Conoce el principio de ALARA o ALADA? en función a los años de experiencia clínica, el grado de instrucción y el lugar donde labora. Se encontró que los profesionales que tienen entre 30 a 40 años de experiencia clínica mostraron una mayor probabilidad de no tener conocimiento sobre el principio de ALARA o ALADA, en comparación con aquellos profesionales que poseen menos de 20 años de experiencia (p = 0,023) (tabla 5). También se encontró que los profesionales con grado de magíster o especialista mostraron una mayor probabilidad de considerar la glándula tiroidea como el órgano que requiere mayor protección, en comparación con aquellos con grados de doctor (p = 0,049).

**Tabla 5:** Análisis de regresión logística de la pregunta “¿Conoce el principio de ALARA o ALADA?” en función a los años de experiencia clínica

Variable	OR	Valor p	IC 95%
20-30 años	1,13	0,855	[0,30, 4,30]
30-40 años	5,20	0,023	[1,25, 21,68]
≥ 40 años	1,81	0,463	[0,37, 8,82]

A la evaluación de la actitud de los dentistas ante los riesgos radiológicos y el uso del CI en radiología bucal y maxilofacial, según sus años de experiencia clínica, grado académico y la entidad donde laboraban, no fueron encontradas diferencias significativas para ninguna de las preguntas realizadas en el cuestionario (p ≥ 0,05). 

Finalmente, con respecto a las preguntas de percepción de los dentistas ante los riesgos radiológicos y el uso del CI en radiología bucal y maxilofacial, se observó diferencia estadística significativa según el grado académico de los dentistas para la pregunta “¿Solo es necesario un consentimiento informado en casos especiales: como niños, mujeres embarazadas y/o pacientes con quimioterapia?”, con un valor de p = 0,001; y según la entidad donde labora para las preguntas “¿Es necesario obtener el consentimiento ante una radiografía bucal y/o maxilofacial?” y “¿Solo se debería informar al paciente acerca de los riesgos mas no necesariamente un documento firmado?”, con un valor de p = 0,07 y p = 0,017, respectivamente. Respecto de los años de experiencia, el cuestionario de percepción no mostró diferencias significativas (p ≥ 0,05).

## DISCUSIÓN

La investigación evaluó el nivel de conocimiento, la actitud y la percepción sobre los riesgos radiológicos y el uso del CI en radiología bucal y maxilofacial entre los odontólogos que laboraban en la UNMSM, según los años de experiencia clínica, grados académicos y la entidad donde laboran. El nivel de conocimiento que se obtuvo fue alto en el 76,40% y medio en el 23,60% de la muestra; no se obtuvo ningún calificativo de bajo. Entre tanto, estos resultados no mostraron diferencia significativa entre las variables testadas (tabla 3). Este resultado difiere con los de Yurt *et al*. ^13^ y Almohaimede *et al*. ^21^, quienes encontraron que el nivel de conocimiento suele ser medio o regular, y que la puntuación de conocimiento disminuye a medida que van aumentando los años de experiencia clínica. Por otro lado, autores como Mahabob *et al*. ^31^ e Ihle *et al*. ^24^ encontraron que el nivel de conocimiento aumenta con los años de experiencia clínica, resultados con los que se concuerda parcialmente debido a que no fueron observados en todos los grupos estudiados. 

Con relación al cuestionario aplicado para evaluar el nivel de conocimiento, actitud y percepción sobre los riesgos radiológicos y el uso del CI en radiología bucal y maxilofacial, el porcentaje de odontólogos que conocían el principio de ALARA o ALADA fue diferente estadísticamente según los años de experiencia clínica. El porcentaje obtenido, del 49,4% (p = 0,041), indica que más de la mitad de la población no conocían estos principios, lo cual es preocupante, puesto que para brindar una protección adecuada del paciente debemos aplicarlos en toda exposición a la radiación. Además, el grupo con 20 a 30 años de experiencia clínica obtuvo un porcentaje de acierto mayor (21,35%) y el grupo de 30 a 40 años de experiencia clínica mostró mayor desconocimiento (24,72%), a pesar de que los principios mencionados tienen una mayor antigüedad de su declaración (tabla 4). Los resultados difieren con los estudios de Mahabob *et al*. ^31^ quienes hallaron que el 70,7% de los encuestados conocían el principio. Almohaimede *et al*. ^21^ y Basheer *et al.*^19^ reportaron que el 68,1% y el 61,8% de sus participantes, respectivamente, conocían el principio de ALARA.

En la literatura se señala a la glándula tiroides como el órgano más sensible a la radiación en la radiografía dental. Al respecto, en la investigación se encontró que el 85,39% de los participantes consideraron que el órgano que requería protección ante una toma radiográfica dental era la glándula tiroidea, siendo significativo según el grado académico (p = 0,038). Este resultado llamó la atención ya que la mayoría de las respuestas correctas fueron de odontólogos con maestría o especialidad, cuando se esperaba que los profesionales con mayor nivel académico fueran los que demostraran este conocimiento. Con todo, los resultados coinciden con los de Ihle *et al*. ^24^, quienes reportaron en su estudio que el 75,8% de los dentistas eligieron la glándula tiroides como el órgano más crítico a proteger durante los procedimientos de imágenes dentales, y son discordantes con el estudio de Yurt *et al*. ^13^, en el cual se encontró que solo el 9,1% de sus encuestados consideró la glándula tiroides como el órgano más sensible a la radiación. 

La pregunta sobre los efectos secundarios en pacientes del uso de radiografías tuvo una respuesta positiva en el 67,42% de los encuestados, pero sin diferencias estadísticas entre las variables estudiadas. Estos resultados son similares a otros, como el realizado por Basheer *et al*. ^19^, quienes encontraron que el 63,5 % de los estudiantes respondieron que las radiografías eran dañinas. Lo mismo ocurren en el estudio de Mahabob *et al*. ^31^, en donde el 89,7% de los participantes creían que los rayos-X dentales eran dañinos. Por su parte, Almohaimede *et al*. ^21^ informaron que el 60,8% de sus participantes sabían que las radiografías dentales eran dañinas para la salud. 

Con respecto al uso de radiografía digital versus la convencional, el 83,15% de los encuestados consideraron que el uso de radiografía digital requería menor radiación que una convencional, pero sin diferencia estadística entre las variables estudiadas. Este resultado es similar al estudio de Mahabob *et al*. ^31^, Yurt *et al*. ^13^, Shanmugam *et al*. ^20^ y Almohaimede *et al*. ^21^, los cuales encontraron que el 73,6%, 83,3%, 58,6%, y el 79,4% de sus participantes, respectivamente, coincidieron en que la radiografía digital necesita menos exposición que la convencional. Este resultado era el esperado, ya que la literatura señala que, además de las radiografías digitales, se debe considerar el uso de películas de velocidad F (la más rápida entre otros tipos) junto con colimadores rectangulares para minimizar la exposición a la radiación ^21^.

Al evaluar la actitud de los participantes ante los riesgos radiológicos y el uso del CI en radiología bucal y maxilofacial, no se observaron diferencias significativas con respecto a la actitud de los participantes según su grado académico, entidad donde laboran y años de experiencia clínica. Sin embargo, podemos resaltar que la mayoría de los participantes usaban siempre o muy frecuentemente delantal de plomo (62,93%) y collar tiroideo (42,70%) en sus pacientes, nuestros resultados son congruentes con su conocimiento de protección radiológica, pues la mayoría de los encuestados consideraron que el órgano que requería mayor protección era la glándula tiroidea. Estos resultados son similares a los obtenidos por Almohaimede *et al*. ^21^, Mahabob *et al*. ^31^ y Anushya *et al*. ^16^, ya que la mayoría de los encuestados usaban delantal de plomo y collar tiroideo. La recomendación de la American Dental Association (ADA) para la protección del paciente ante una exposición radiológica es usar collares tiroideos siempre que sea posible, mientras que el uso del delantal de plomo puede no ser necesario ^24^. 

Cuando se evaluó la actitud de los participantes con relación a si se ubican detrás de una barrera de plomo durante la exposición, el 56,18% señaló que siempre o muy frecuentemente lo hacían, mientras que el 12,36% nunca se paró detrás de una barrera de plomo durante la exposición. Estos resultados concuerdan con los de Anushya *et al*. ^16^, quienes encontraron que el 70,5% de los dentistas se pararon detrás del escudo de plomo y el 74% se mantuvo a una distancia de 6 pies de la radiación. En el estudio de Ihle *et al*. ^24^ se halló que las tres cuartas partes de los dentistas informaron como protocolos de precaución el permanecer detrás de una barrera protectora durante la exposición a la radiación. Solo dos tercios de los dentistas se colocaron detrás de una barrera protectora. Por otro lado, con respecto a la ejecución de la técnica radiográfica intrabucal, el 20,22% de los participantes mantenían la película radiográfica en la boca de los pacientes durante la exposición. Este resultado es similar al de Javali *et al*. ^17^, quienes hallaron que el 17,5% de los odontólogos estabilizaron por sí mismos la película radiográfica, y al de Ilgüy *et al*. ^32^, donde el 16,8% de los odontólogos señaló que ayudaba a sostener la radiografía en la boca del paciente durante la exposición. Este resultado es preocupante ya que, a pesar del alto conocimiento que presentan los participantes con respecto a protección radiológica, aún persisten protocolos de ejecución inadecuados. Esto puede deberse a que consideran que las dosis utilizadas son muy bajas; sin embargo, no se ha considerado el riesgo de dosis acumulativas por exposición continuada ni la probabilidad de sinergia con otras radiaciones a las que pueden estar sometidos como resultado de tratamientos en otros campos médicos, lo cual sería un factor importante en la evaluación de la dosis acumulada absorbida. 

Con respecto al uso del CI, el 37,08% y el 26,97% de los participantes señalaron que siempre y frecuentemente hacían uso de este en su práctica clínica diaria, respectivamente. Este resultado difiere con el estudio de Nono *et al*. ^33^, los cuales encontraron que, a pesar de que el 85,3% de los odontólogos obtuvo el consentimiento de los pacientes antes de comenzar cualquier procedimiento, solo el 5,3% obtuvo el CI por escrito de los pacientes. En otra pesquisa en Nigeria ^34^, el 61,6% de los dentistas logró el consentimiento por escrito de sus pacientes, y Kotrashetti *et al*. ^35^ encontraron que el 63,6 % de los dentistas también lo obtuvo. 

El 34,83% y el 28,09% de los participantes siempre y frecuentemente informan a los pacientes acerca de los riesgos de la radiación antes de tomar una radiografía en su práctica diaria. Estos resultados difieren con el estudio de Javali *et al*. ^17^, en donde sa halló que el 51% de los dentistas no tenían experiencia explicando los riesgos/beneficios de la radiación a los pacientes. Los profesionales deben explicar el plan de tratamiento a los pacientes y estos deben ser informados de los posibles resultados adversos del tratamiento, ya que tienen derecho a rechazar la intervención propuesta ^17^.

Al evaluar la percepción de los participantes ante los riesgos radiológicos y el uso del CI en radiología bucal y maxilofacial, se encontró que el 48,31% de los participantes estuvieron de acuerdo y el 38,21% estuvieron totalmente de acuerdo en que se debería informar al paciente acerca de los riesgos de cáncer causados por radiación. Estos resultados fueron similares a los de Younger *et al*. ^10^, quienes encontraron que la mayoría de los radiólogos estuvieron de acuerdo en que es importante informar a los pacientes acerca de los riesgos causados por radiación ^2^. En la actualidad, los pacientes tienen relativamente poca información sobre los peligros radiológicos; no obstante, tienen una gran necesidad de información, sobre todo después de conocer los peligros de desarrollar un cáncer por radiación, lo que les hace ser más conscientes de la necesidad de un CI ^12^. Adicionalmente, se observó que el 31,46% de los participantes en la presente investigación están de acuerdo en que solo se debería informar al paciente acerca de los riesgos involucrados en un registro radiográfico, pero que no es necesario un documento firmado, en igual proporción (31,46 %), los participantes consideran estar en desacuerdo con el enunciado. Estos resultados son similares a los encontrados en Javali *et al*. ^17^, quienes encontraron que el 51% de los dentistas le explicaron al paciente los riesgos/beneficios de la radiación antes de la exposición radiográfica, pero el 56,5% no realizó el CI antes de adquirir la radiografía. El 30,34% de los participantes están de acuerdo en que solo es necesario realizar un CI en radiología bucal y maxilofacial en casos especiales como niños, mujeres embarazadas y/o pacientes con quimioterapia. La percepción del odontólogo ante esta pregunta fue estadísticamente diferente (p = 0,001) con respecto al grado académico de los participantes. Estos resultados difieren de los de Portelli *et al*. ^8^, los cuales encontraron que casi todos los radiólogos (97,6%) opinaron que los pacientes pediátricos y/o sus padres/tutores deberían recibir información sobre los beneficios y riesgos de un examen médico por imágenes. 

El presente estudio tuvo algunas limitaciones. En principio, se realizó en una sola institución, por lo que es difícil generalizar los hallazgos más allá de la muestra del estudio. Además, si bien las respuestas proporcionadas se basan en una representación satisfactoria de odontólogos, no se pudo controlar el número de encuestados y este varió entre especialidades y sectores de trabajo, por lo que la distribución no fue igual entre los participantes. También existen limitaciones relacionadas con el uso de cuestionarios, por lo cual, aparte de la posibilidad de que algunas respuestas pueden modificarse por el factor memoria, es posible que los participantes no hayan proporcionado una respuesta veraz de lo que realmente dicen o hacen en su práctica diaria. Sin embargo, se espera que esto sea bastante improbable ya que se aseguró el anonimato de todos los participantes.

## CONCLUSIÓN

Los odontólogos que laboran en la Facultad de Odontología de la UNMSM tuvieron un buen conocimiento, actitud y percepción sobre los riesgos radiológicos del uso de CI, y en su mayoría no fueron influenciados por factores como el grado académico, entidad donde laboran y años de experiencia clínica. Aunque el estudio se focaliza en una sola institución, esta se puede considerar como un referente nacional en la formación de odontólogos en el Perú. Los resultados obtenidos ofrecen una mirada representativa del conocimiento, las actitudes y las percepciones que podrían estar presentes en otros entornos educativos y clínicos, los cuales contribuyen a generar conciencia en los odontólogos, promover el uso del CI en radiología odontológica, así como orientar intervenciones formativas, políticas institucionales y marcos normativos.

## Financiación:

Este estudio no recibió ninguna financiación específica.
